# Perylene-Based Chromophore as a Versatile Dye for Light Amplification

**DOI:** 10.3390/ma15030980

**Published:** 2022-01-27

**Authors:** Alina Szukalska, Adam Szukalski, Justyna Stachera, Dorota Zajac, Ewa Chrzumnicka, Tomasz Martynski, Jaroslaw Mysliwiec

**Affiliations:** 1 The Advanced Materials Engineering and Modelling Group, Faculty of Chemistry, Wroclaw University of Science and Technology, Wybrzeze Stanislawa Wyspianskiego 27, 50-370 Wroclaw, Poland; adam.szukalski@pwr.edu.pl (A.S.); dorota.zajac@pwr.edu.pl (D.Z.); jaroslaw.mysliwiec@pwr.edu.pl (J.M.); 2 Faculty of Materials Engineering and Technical Physics, Poznan University of Technology, Piotrowo 3, 60-965 Poznan, Poland; justyna.m.stachera@doctorate.put.poznan.pl (J.S.); ewa.chrzumnicka@put.poznan.pl (E.C.); tomasz.martynski@put.poznan.pl (T.M.)

**Keywords:** liquid crystal, stimulated emission, polymer, hybrid systems, organic, perylene

## Abstract

One of the challenges for modern optoelectronics is to find versatile, easily adaptable components for novel laser-based technologies. A very attractive perylene-derivative chromophore in different organic matrices for high-performance light amplification is discussed and outlined. Our approach demonstrates the outstandingly compatible laser dye and a viable strategy to provide an effective optical gain for stimulated emission enhancement. Through structural control, we produce simple optical devices embedded in organic matrices, such as poly(methyl methacrylate), nematic liquid crystalline (NLC) mixture, and a hybrid emulsion system (poly(vinyl alcohol) PVA + NLC mesophase). Importantly, we investigate and compare the spectroscopy of differently constructed organic systems in terms of stimulated-emission thresholds and light amplification process efficiency. Moreover, we report the effects of tunability for LC cells by an applied external electric field stimulus. Future directions of laser systems are outlined with an emphasis on the role of the perylene derivative. The studies meet current challenges in the field of modern organic technologies dedicated to various optoelectronic systems, including touch screens, displays, and Li-Fi networks.

## 1. Introduction

In recent years, there has been extraordinary interest in varied materials dedicated to light amplification (LA) and subsidiary laser-based studies. Modern and newly synthesized compounds acquired for stimulated emission (STE) enhancement have to meet certain requirements, primarily the following: high quantum yield [1], long lifetime [2], low STE threshold [3], and remarkable tunability [4]. The fact that organic materials are still commonly used and desired for this kind of application can be surprising. In the age of inorganic devices providing excellent photostability and efficiency (such as semiconductor perovskite dyes or quantum dot LEDs [5,6,7,8,9]), the role of organic materials can be erroneously seen as limited. However, in comprehensive studies, many research groups have revealed significant changes in the creation and processability of hybrid devices [10,11]. The decent majority of organic materials show valuable optoelectronic properties and can be easily combined to multiply their excellent advantages. A key requirement they provide is the large stimulated emission cross-section and high spectral tunability as a consequence of the easy material connection process [12]. The excellent broadband photoluminescence can be obtained by the well-planned material engineering aimed at the synthesis of a group of organic luminescent compounds (e.g., pyrazoline derivatives) [13]. This strategy has undoubted advantages; namely, it has been proven that the pyrazoline derivatives with highly polar end-groups present large dipole moments, which have a positive effect in the generation of second-order nonlinear optical effects. Due to their chemical structure, some of the derivatives show the effect of amplified spontaneous emission (ASE). It is clearly presented that with the well-planned synthesis process and attaching appropriate chromophore groups, different colors of fluorescence and stimulated emission effects can be obtained.

Soft-matter organic compounds such as liquid crystals (LCs) or polymers doped with luminescent dyes can be exploited in complex, micro, or macro scale systems according to their mechanical flexibility, good rheological features, and easy and cheap device fabrication process [14,15]. Other benefits of such spectroscopic systems are the high-quality color reproduction, sensing properties, ability to emit light within the full color gamut, excellent control of their tone, and white lasing enhancement [16,17,18,19,20,21]. The operational principles of organic lasing systems make them very appealing for the new generation displays [22] (including laser-powered ones), projectors, mobile multimedia, lighting technologies, sensors [23], and wireless communication [24] and provide a further step towards modern optoelectronics.

Recalling the hybrid organic devices designated for light amplification, it is necessary to gain insight into materials’ composition and role. The systems that exploit the stimulated emission phenomenon require a conveniently conformed optical gain, which is a basic principle for the operation of all lasers. Luminescent dyes offer a huge number of potential applications; therefore, their search and synthesis are a current as well as emerging topic. As an example, the well-known and widely reported chromophores, such as rhodamine B for high-signal, laser-irradiated detectors [25], were presented. Many routes for electrochromic, sensing, and displaying technologies are currently open, according to the presentation of photophysical properties of newly synthesized dyes, such as pyrene-BODIPY compounds [26]; panchromatic [27], solvatochromic, and halochromic tricarbocyanine; and heptamethine cyanine chromophores [28]. The outstanding properties of laser technologies and solar cells, such as high quantum yield, great solar to electrical energy conversion efficiency, high molar extinction coefficient, and light absorption in a visible range of the spectrum, are in high demand [29,30].

The perylene-based dyes (especially perylene diimides (PDIs) and perylene tetracarboxylic acid dianhydride (PDA)) are widely reported in the literature and practically used as high-performance pigments [30,31]. As strong electron acceptors that show high luminescence performance, PDIs are commonly utilized in solar cells with polymer bulk heterojunction (BHJ [32]) and have become a reference model for newly discovered compounds. Among their many applications, these materials are used to form solar concentrators, fluorescent sensors, novel technology OLED devices [33]. PDIs find application as laser dyes due to their high luminescence efficiency [34]. According to the literature [35], PDIs can be considered as very useful materials due to the versatility of their chemical structure. Solubility, tunability, electronic, optical, and morphological features for PDIs may vary depending on the substituents in the ortho, bay, or imide positions. For example, substituents on the imide nitrogen atoms can improve the solubility of PDIs [36]. PDA is well known as a semiconductor material according to the π–π conjugation observed between the layer-stacked molecules. This material provides great electrical properties but also is well known due to its good optical features [37]. In this work, we focus our attention on the attractive perylene-derivative named 3,4,9,10-tetra(heptyloxycarbonyl)perylene (abbreviated here as PER). The molecular environment of the examined perylene dye is different from that in PDIs and PDA. We chose this chromophore for our studies according to the reported good solubility in liquid crystalline matrix provided by the esters of alcohols having 5–7 carbon atoms [38]. The selection of the perylene tetracarboxylic derivative for research is based also on the fact that non-imide derivatives have high fluorescence quantum yield (QY) from singlet states. They have a lower tendency to aggregate in solutions as well as in Langmuir–Blodgett layers, thus maintaining a high QY. Their solubility is primarily related to the length of the alkyl chains and not to whether they are imide or non-imide derivatives. They show good solubility in liquid crystals, and above all, aggregates appear at high (5 × 10^−2^ mol/dm^3^) concentrations [39,40,41,42]. It is also worth mentioning that during the research it turned out that also the aggregated form shows stimulated emission and has undoubted advantages, ensuring great photostability [42]. For our studies, it is crucial to find a versatile laser dye, not only in terms of its solubility but especially for the stimulated emission performance. PER serves as the perfect chromophore, according to the fact that it can be effectively pumped and obtain the population inversion in three different matrices: Poly(methyl methacrylate) (PMMA, liquid crystalline 5CB, and the mixture named 1742). This feature is very unique in the context of organic dyes, as it is often found that quenching of the stimulated emission depends on the matrix or even the solvent used. Other important parameters were the successful research on their absorbance and fluorescence in both nematic and isotropic states as well as the good photostability [42]. We chose a derivative with seven carbons in aliphatic chains due to its higher lability and lower tendency to aggregate compared to a derivative with shorter chains. Our objective was to clarify the role and influence of the various organic matrices utilized and the laser dye in the stimulated emission phenomenon nature. The effect found leads to the accurate description and comparison of STE performance, specifically the applied external electric field significance, the designation of energy thresholds, and spectral profiles in different organic matrices (nematic LCs, poly(methyl methacrylate) (PMMA), and hybrid emulsions containing poly(vinyl alcohol) (PVA)). The adoption of diverse organic materials allows for the creation of forthcoming practical appliances containing the laser dye selected and investigated herein. Moreover, it opens up prospects for exploiting the excellent properties of the perylene derivative in terms of emitting enhanced light. The optical gain achieved in the proposed systems can be beneficial for the development of novel optoelectronic devices, such as new-generation displays, highly flexible optical sensors, or bendable microlasers. 

## 2. Materials and Methods

### 2.1. Synthesis of PER

The synthesis process was performed at the Lodz University of Technology (Poland). The method of producing new fluorescent dyes, derivatives of 3,4,9,10-tetra(heptyloxycarbonyl)perylene, is described in [38,39,40]. Tetramethyl ester of perylene-3,4,9,10 tetracarboxylic acid was subjected to alcohol transesterification in the environment of excess alcohol at the temperature of 80–160 °C. The duration of the process was between 3 and 60 h in the presence of sodium alkoxide as a catalyst. At the same time, the evolving methanol was distilled out of the reaction medium. After completion of the reaction, activated carbon was added to the medium and filtered, and a part of the solvent was distilled off from the filtrate and left for crystallization. Then, the crystallized dye was filtered off and dried at 20–60 °C under normal conditions or under reduced pressure. The dyes obtained by the process showed strong fluorescent properties in organic solvents as well as in liquid crystal cells. The structures of the dyes were confirmed by the use of the two methods—NMR and MS spectra. In order to estimate the purity of the synthesized compounds, the thin-layer chromatography (TLC) method (on silica gel 60 F254 Nano DC plates, with acyclohexane/dioxane 4:1 mixture as the eluent) was used.

### 2.2. The ^1^H, ^13^C, and DEPT-135 NMR Spectra Measurements

The ^1^H, ^13^C, and DEPT-135 NMR spectra were measured on a Bruker 600 MHz Avance II NMR spectrometer (Bruker Biospin Corp. Billerica, MA, USA). The compound was dissolved in deuterated chloroform (CDCl_3_) for the NMR experiment. The chemical shift values were reported on δ scale in ppm, relative to TMS (δ  =  0 ppm) and CDCl_3_ (δ_H_ 7.28 (s) and δ_C_ 77.01 (t)). The measured spectra can be found in Supplementary Data as Figures S1–S3.

^1^H-NMR (600 MHz, CDCl_3_), δ (ppm): δ 8.30 (d, *J* = 7.8 Hz, 4H), 8.06 (d, *J* = 7.8 Hz,4H), 4.34 (t, *J* = 7.2 Hz, 8H), 1.84–1.79 (m, 8H), 1.57 (s, 4H), 1.49–1.44 (m, 8H), 1.42–1.37 (m, 6H), 1.34–1.32 (m, 14H), 0.92 (t, *J* = 6.9 Hz, 12H).

^13^C-NMR (151 MHz, CDCl_3_), δ (ppm): δ 168.49, 133.09, 130.51, 130.42, 129.04, 128.85, 121.38, 65.65, 31.74, 29.02, 28.61, 25.98, 22.60, 14.02. 

^13^C-DEPT-135 NMR (600 MHz, CDCl_3_), δ (ppm): δ 130.42 (Ar-CH), 121.38 (Ar-CH), 65.65 (CH_2_), 31.75 (CH_2_), 29.02 (CH_2_), 28.61 (CH_2_), 25.99 (CH_2_), 22.61(CH_2_), 14.07 (CH_3_).

### 2.3. Polymer Matrix

The steps of device realization were as follows: The PMMA powder was dissolved in dichloromethane (DCM) solvent at a concentration of 5%, creating the matrix for hydrophobic PER dye. Aforesaid homogeneous solution (PER concentration varying between 0.5%, 1.0%, 2.0%, and 3.0% *w/w*) was poured onto a basic microscope glass, using the drop-casting deposition method (the same volume of solution in all cases). The samples were dried under the same DCM solvent atmosphere.

### 2.4. Liquid Crystalline Matrix

We present the measurements obtained on two LC cells with different geometry. PER was incorporated into the 1742 nematic mixture with negative dielectric anisotropy Δε (purchased at the Military University of Technology, Warsaw, Poland). We used a homemade LC cell with homeotropic alignment imposed by the use of an orienting polymer. An ITO transparent electrode was used in order to prepare the cell for measurements using an electric field. The LC gradient cell (LCGC), characterized by a perylene concentration of 1.5 × 10^−2^ mol/dm^3^ and varied thickness, was obtained by use of two different-height spacers (10 μm and 60 μm). In the second LC cell, with a constant thickness of 20 μm, the perylene concentration was 1.0 × 10^−2^ mol/dm^3^. The prepared host–guest mixture was incorporated into the LC cell by capillary forces.

### 2.5. Emulsion Environment

The idea behind this experiment was to form hydrophobic LC droplets doped with PER dye and suspended in poly(vinyl alcohol) dissolved in distilled water (hydrophilic matrix). For the stimulated emission test, the droplets thus produced were regarded as optical resonators, varied by the time of the homogenization process. The idea was to create a system on a widely referenced liquid crystal called 5CB, as it has already been successfully tested to create an emulsion with other laser dyes [43]. The 5CB matrix was also chosen to demonstrate the diversity of matrices that can be used to create host–guest systems with the perylene derivative presented here. After the optimization process, the 5CB matrix was doped with PER dye in a concentration of 5 mg/mL, providing sufficient conditions for stimulated emission enhancement, supported by the previous experiments referred to in the literature [44]. We utilized the PVA/H_2_O in a concentration of 7.5% *w/w*, based on the literature [44]. This prepared polymer matrix is characterized by adequate viscosity to keep the droplets separated from each other. Moreover, the 7.5% concentration of PVA ensures the perfect conditions for the homogenization process and the formation of a good-quality droplet shape, and it dries in the air in a relatively short time for water (maximum one day). The ratio of LC-PER mixture to PVA matrix was 30 μL per 1 mL, respectively, and was constant for all samples.

### 2.6. Spectroscopic Measurements

The absorbance spectra were recorded with the help of the Jasco V-670 spectrophotometer.

The fluorescence spectra were acquired with the use of Hitachi F-4500 spectrofluorometer (λ_exc_ = 440 nm).

For the STE enhancement as the source of laser light, the third harmonic of Surelite II Continuum nanosecond pulsed Nd:YAG laser with the repetition rate of 10 Hz and pulse duration time of 6 ns was used. The laser source is suitable for STE experiments in organic materials since it provides pulse operation without continuously heating the sample. Thanks to this, it is certain that the measurement was performed for the nematic (not the isotropic) phase. All measurements were averaged (10 pulses for each measurement) to make sure that the appropriate, reliable amount of data was presented and that the sample was not overheated or overused. The experimental setup consisted of Optical Parametric Oscillator (Horizon, high-efficiency mid-band OPO by Continuum), providing the possibility of choosing desired wavelength (λ = 480 nm). The laser light was driven to the half-wave plate, passed the polarizer systems, and finally formed a stripe geometry with a length and height of 4 mm and 0.5 m, respectively. 

For the applied electric field experiment, the electricity was conducted to the sample with the help of two electrodes connected with a DC power supply. The experiment was for the LC cells above the stimulated emission energy threshold in both cases (9.5 mJ/cm^2^ for LCCC and 9.7 mJ/cm^2^ for LCGC).

## 3. Results and Discussion

### 3.1. Polymer Matrix

**Figure 1:**
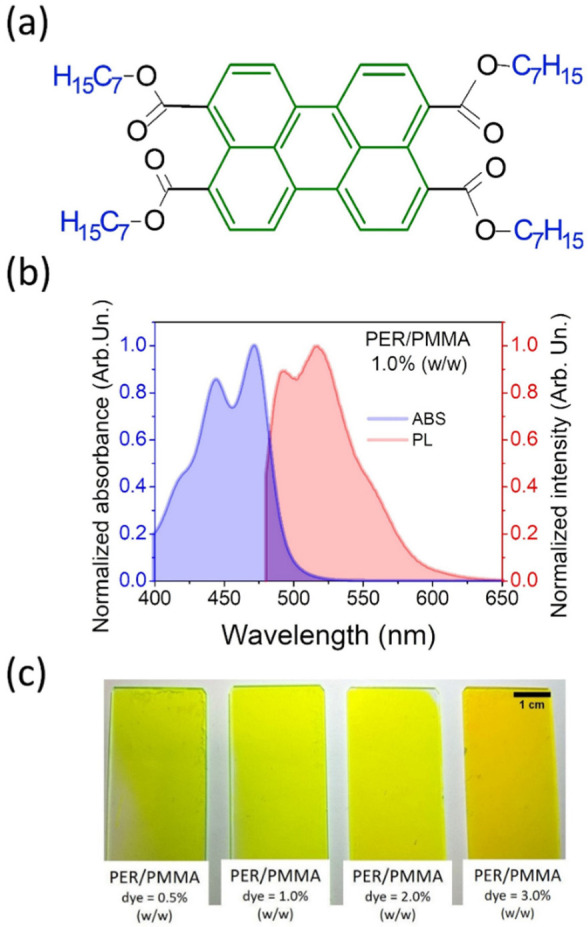
(**a**) Molecular structure of 3,4,9,10-tetra(heptyloxycarbonyl)perylene (PER) with perylene core and four aliphatic ligands marked in green and blue, respectively. (**b**) Absorbance and photoluminescence spectra of sample PER/PMMA (1% *w/w*). (**c**) The photograph presenting set of prepared thin films with various perylene dye concentrations (0.5%, 1.0%, 2.0%, and 3.0% *w/w*); scale bar: 1 cm.

As the first type of the investigated hybrid devices, we present the one composed of PER introduced into the PMMA matrix. The chemical structure of the chromophore is given in Figure 1a. 

The absorbance and emission spectra present complex structures; there are visible oscillating rotational bands and an additional band coming from aggregates. Two maximal absorbance peaks are seen, one at λ = 472 nm and the weaker one at λ = 444 nm (Figure 1b). The fluorescence maxima are placed at λ = 517 nm and at λ = 493 nm, which indicates that light emission is in a wide range from blue-green to green region. The set of prepared samples is presented in Figure 1c, as the macro photographs [38,39,40,41,42].

**Figure 2:**
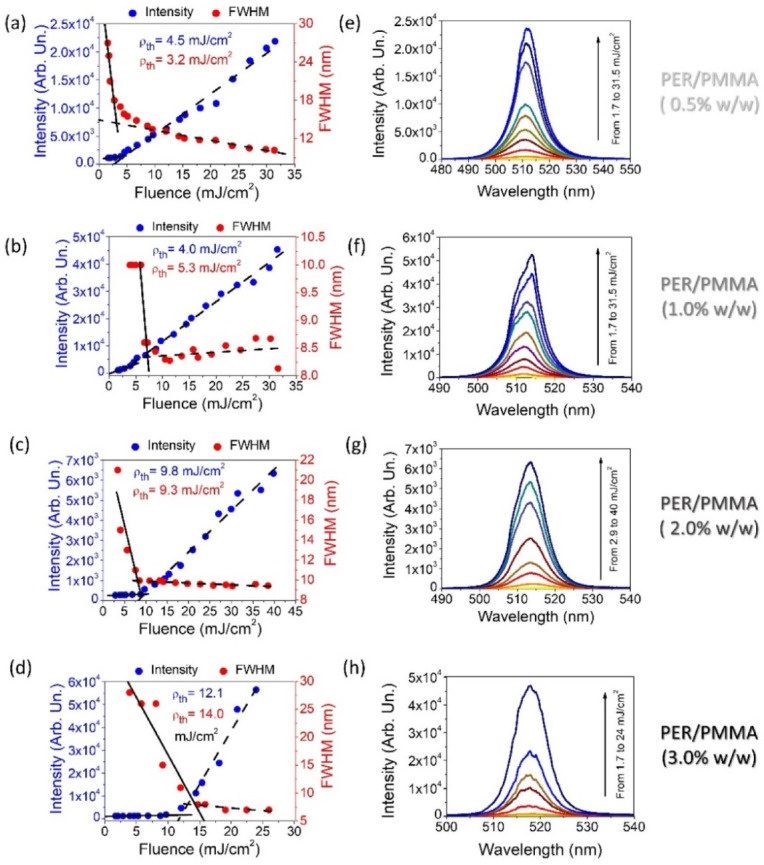
PER/PMMA system—the stimulated emission energy threshold designation for all dye concentration samples (**a**–**d**). Pump laser beam shape formed to stripe with length and height at 4 mm and 0.5 mm, respectively. Stimulated emission spectra vs. I_pump_ for all samples (**e**–**h**).

The next step was to examine all prepared samples in the context of STE enhancement. The samples functionalized with the PMMA matrix were tested to gain information about their STE energy threshold ($\rho_{\mathrm{th}}$) values. As it is indicated in Figure 2a–d, STE ($\rho_{\mathrm{th}}$) was defined with the use of two methods. The first one relies on the full width at half maximum (FWHM) parameter change monitoring. The second one, called the light-in–light-out technique (LI-LO) here, is based on the control of changes in the integrated intensity of measured spectra. These values collected before and after the characteristic inflection were fitted with a linear function. Such a presentation of the results allows the energy threshold of stimulated emission to be revealed. Both methods coincide with the results and were utilized to obtain information about the STE $\rho_{\mathrm{th}}$ in the most accurate way.

Figure 2a–d highlights a significant trend. Namely, with the increase in the dye concentration, a rise in the energy threshold value can be observed as well. In the sample characterized by the dye concentration of 0.5% *w/w*, clear spectrum narrowing was found at 3.2 mJ/cm^2^ (for the FWHM method). The energy threshold, indicated with help of LI-LO characterization, was noticed for pumping fluence of 4.5 mJ/cm^2^. The sample of 1% PER concentration in the PMMA matrix shows the FWHM and energy density thresholds of 5.3 and 4.0 mJ/cm^2^, respectively. Significantly, 2.0% *w/w* PER-doped PMMA creates a sample with the $\rho_{\mathrm{th}}$ value of nearly 9 mJ/cm^2^, and here the good compatibility of both methods is noticed. In the most concentrated sample (3%), characteristic deflection of the trend line is visible for the energy density of 14 mJ/cm^2^, and a significant reduction in the half-width can be noticed for 12 mJ/cm^2^. Surprisingly, referring to the literature, we could anticipate the opposite situation. Indeed, perylene dyes and their derivatives are very attractive for J-aggregate formation possibility [45,46]. However, this situation is successfully observed mostly in the case when perylene derivatives (especially bisimides) form a liquid crystalline columnar phase or in the host–guest systems with the LC matrix [42,47,48,49,50]. Therefore, the chance of aggregate-induced emission (AIE) observation in PER materials is high, although in the considered case the effect was not found. There is a possible explanation for this phenomenon. It assumes the role of the matrix in the formation of aggregates. The LC mesophase, according to its fluidity, creates a convenient environment for the creation of aggregated forms or crystals of dissolved dyes. According to the second major feature of LCs (namely long-range molecular ordering plus anisotropy), the orientation of the guest molecules to the axis of preferred solvent alignment is expected. These factors together create perfect conditions for the formation of aggregates, also characterized by anisotropy. Furthermore, the molecular arrangement is reflected in the analysis of optical and spectroscopic properties. It is difficult to find similar properties with the use of a polymer matrix, which is stiffer than LCs and restricts the freedom of growing aggregated regions. The spectra obtained during the stimulated emission experiment are correlated with the designated energy thresholds and can be found in Figure 2e–h. The maxima of emitted light in all samples are localized at about 515 nm and are indicated using cyan-green color. 

### 3.2. Liquid Crystalline Matrix

**Figure 3:**
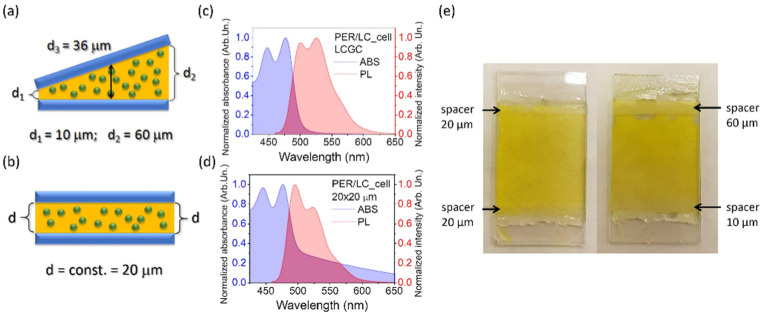
The schematic presentation of prepared LC cells. The thickness of the sample is a changing gradient (from 20 to 60 μm), where a black arrow indicates a region where absorbance, emission, and STE measurements were performed (**a**), and constant (20 μm) (**b**). Absorbance and photoluminescence spectra were collected for liquid crystalline cells doped with perylene dye for the gradient sample (**c**) and the 20 μm thick sample (**d**). The photograph presents the macroscopic morphology of all investigated LC systems in the cell form (**e**).

The PER dye, according to its considerable ability to serve as a guest in LC-based systems, was used in order to produce three different liquid crystalline cells. The experimental details of the first system together with the description of the STE phenomenon and spectral characteristics, including its photostability, have already been presented elsewhere [42]. It is worth mentioning the experimental result showing that PER in the LC nematic environment, under the conditions described in [42], does not show any photodegradation after 12,000 laser pulses (with an energy density equal to 5.9 mJ/cm^2^), which is an outstanding effect compared to other organic materials. In this contribution, we focus our attention on another two LC cells. The scheme of the first one is presented in Figure 3a. The LC gradient cell (LCGC) is characterized by a varied thickness, obtained by the use of two different-height spacers (d_1_ = 10 μm and d_2_= 60 μm width). The arrow on the scheme indicates the area of STE measurement, and it is equal to d_3_= 36 μm. Further discussion about the gradient LC cell spectroscopy investigations is presented in the Electronic Supplementary Information (ESI) file (Figure S4).

The second LC cell was arranged with a constant thickness (liquid crystalline constant cell (LCCC)) of 20 μm (Figure 3b). Both LC cells were subjected to the measurement of basic spectroscopic properties, namely absorbance and fluorescence (Figure 3c and Figure 3d for LCGC and LCCC, respectively), which seem to be similar to the PER/PMMA systems considered before. The absorbance and emission spectra show visible oscillating rotational bands and an additional band coming from aggregates. The differences in spectra presented in Figure 3c,d arise from the aggregation of PER in liquid crystal matrices. The set of the prepared LC cells is presented in Figure 3e. 

**Figure 4:**
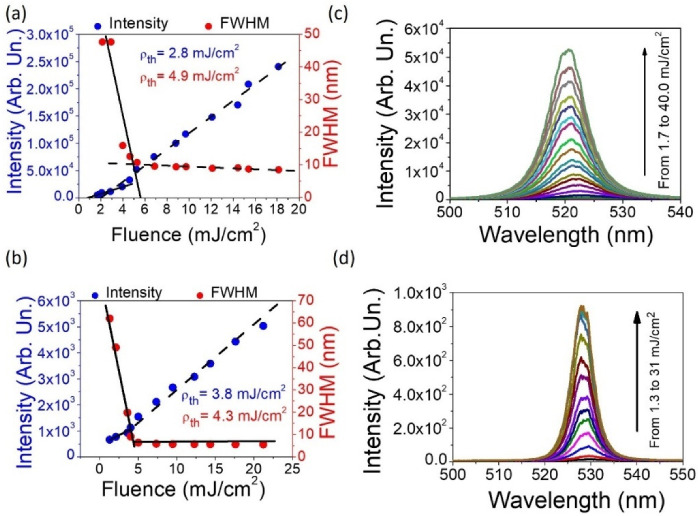
The stimulated emission energy threshold values obtained for the LC cells with gradient thickness (10 × 60 μm) (excited in the central part of the sample, correlated with the thickness of 36 μm) (**a**) and constant thickness (20 μm) (**b**). The STE spectra acquired for gradient (**c**) and constant (**d**) thickness LC cells pumped with various levels of laser energy.

The STE experiment (Figure 4a,b) provides information about the similar STE thresholds for both examined samples. The slightly lower average STE threshold obtained in the LCGC sample (4a) (2.8 mJ/cm^2^ for LI-LO method and 4.9 mJ/cm^2^ for the FWHM, averaged = 3.85 mJ/cm^2^) than in the LCCC sample (3.8 mJ/cm^2^ and 4.3 mJ/cm^2^ for both methods, respectively, and average = 4.05 mJ/cm^2^). It is commonly accepted that the lower energy thresholds of used materials [51,52] are usually obtained in thicker active layers according to the longer optical path of light amplification as well as more favorable waveguiding conditions. The higher-quality optical materials can significantly reduce the optical gain energy threshold value due to the lower light emission losses. However, in this case, both cells are composed of the same materials and differ in the geometry of the samples and the dye concentration. It is worth noting that the slight threshold changes may be due to thickness differences, but additional statistical tests should be performed to confirm the tendency.

The spectral profiles obtained during the experiment are presented in Figure 4c,d. The emitted light is green in color (maximum about 520 nm). Therefore, the use of LC host for the PER guest can lead to a slight change in color of the emission towards green, compared to the system where PMMA serves as the matrix. 

**Figure 5:**
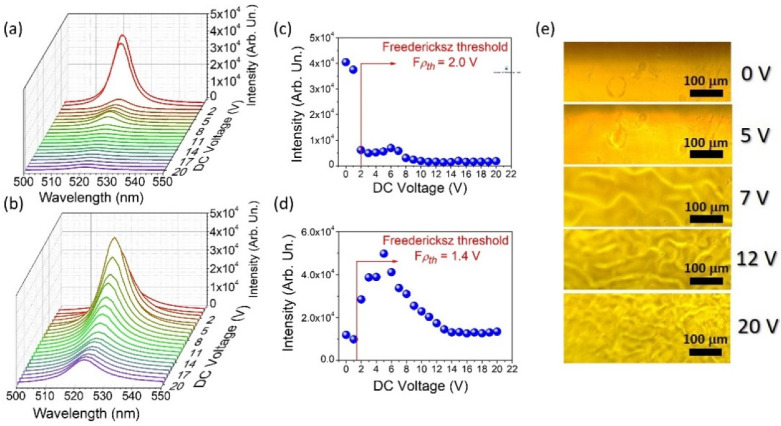
Applied DC voltage dependence of the induced output light intensity measured for the LC cells characterized with a gradient thickness (**a**) and a thickness of 20 μm (**b**). Freedericksz threshold values were estimated for both samples ((**c**) and (**d**), respectively). Microscopic images presenting the structural changes of gradient LC cell surface during the DC voltage application (**e**).

LC matter in the context of light amplification is known in the literature according to its fascinating tunability properties. Fluidlike behavior and long-range orientational molecular order make LCs very susceptible to applied external fields. In the group of successively used external stimuli, it is worth distinguishing the electric and magnetic fields, environmental temperature changes, and mechanical stress. For the prepared LC cells, the electrical control was implemented to understand whether the differences in enhanced emission characteristics could be noticed. As is presented in Figure 5a, the intensity of emitted light in the LCGC sample rapidly increased at the point corresponding to 1.4 V application. 

The corresponding plot presenting the intensity as a function of applied DC voltage (Figure 5c) clarifies the observed situation by the indication of the Freedericksz threshold value. This point should be understood as the crucial moment for the molecular transition in the LC matrix, which strongly influences the light amplification conditions. In this instance, the LC cell geometry designed for the experiment described here should be recalled. Both samples show a homeotropic alignment, imposed by the method of assembly. It is characterized by long axes of LC molecules aligned perpendicularly to the surface of the substrate (here, glass slides). Another important feature is that the LC mixture here presented shows a negative dielectric anisotropy (∆ε = −3.250), and in this case, while measured at low frequencies, the dipole moment is localized perpendicularly to the long axes of the liquid crystalline molecules. A consequence of such alignment is reflected in the fact that the molecules above the Freedericksz point tend to align their long axes perpendicularly to the electric field direction. Therefore, the dipole moment of each molecule was set parallel, as was the polarization state of excitation laser light. From theory, such a situation provides optimum conditions for excitation of the luminescent/laser dye and light propagation. It could be observed in the emission intensity as a strong increase. Exactly this effect can be observed in the case of LCGC (Figure 5a,c) above the Freedericksz threshold indicated here (F$\rho_{\mathrm{th}}$ = 1.4 V). After the application of very low voltage (merely 6 V), the STE output intensity increased 5-fold. With such an irrelevant value of the applied voltage, a five times greater light amplification can be achieved. Referring to the tendency presented in the Freedericksz threshold analysis (Figure 5c), to realize a similar effect with no application of electric field, it would be required to increase the energy density to about 40 mJ/cm^2^. Therefore, the energy used for optical pumping would be four times higher. Certainly, it can be said that the electric field application is much more energy-efficient. In Figure 5e, one can compare the LC surface changes under the DC voltage application for the LCGC sample. From the point referring to the 7 V application, the “boiling liquid” effect is visible. In the LC cell, one can distinguish the turbid movements induced by the electric field, which leads to the formation of liquid crystalline domains. Noticeable wave-shaped objects (7 V application) turn into more concentrated, oval LC centers (12 V), and finally, they form tiny structures of constantly migrating and shape-changing areas. Together with the creation of LC domains, the light amplification magnitude successively drops to the moment (corresponding to 14 V application) in which it reaches almost the initial value. Such behavior can be explained in the terms of strong hydrodynamic instabilities induced by ionic current flow and is reflected in the interesting phenomenon called dynamic light scattering (DLS) [53,54]. The nature of this process can be seen in a significant increase in turbulent flows in the LC materials when a hydrodynamic momentum connected with a shear flow exceeds the momentum of the electric field. This phenomenon can be observed in the LC materials characterized by low dielectric anisotropy, as in this case [54]. The disturbances in the LC cell lead to the creation of a chaotic molecular arrangement and less effective conditions for constructive light scattering, which results in a reduction in the emission intensity. This is an excellent example of how the liquid crystalline matrix allows controllable, easy, and low-energy tuning by the electric field application and cell construction. In the case of LCCC, such a tendency can be observed as well. A surprising difference in the Freedericksz threshold value is noticed. For LCGC, this point refers to a value of 1.4 V; for LCCC, it is still low but is about two times higher (3 V). The effects observed on the two investigated LC cells, made of the same materials and obtained with the same homeotropic molecular construction, theoretically should be similar in their features. Once again, it is worth recalling the main property differing between both cells—thickness. Indeed, in the cells with a thinner active layer, much greater forces of molecular anchoring can be observed and are associated with their contact with the orienting surfaces. According to the literature [55], LC molecules in the close presence of glass walls are less likely to rotate effectively in response to the influence of external stimuli. In thin layers, the population of molecules arranged in this way is proportionally more significant than those in the center, rotating smoothly and freely. Therefore, the efficient and optimal rotation of the molecules in the LCCC cell dominated the stimulated emission response at the point of 3 V, where the intensity increase was noted. Moreover, we can observe the nearly five times higher intensity in comparison with the 0 V application, which proves that it is possible to obtain effective electric field tunability in LC cells differing in thickness and geometry. The whole experiment presents the undeniable potential of designing PER-LC-based cell devices for the achievement and control of the stimulated emission process and its optical features. 

### 3.3. Emulsion Environment

**Figure 6:**
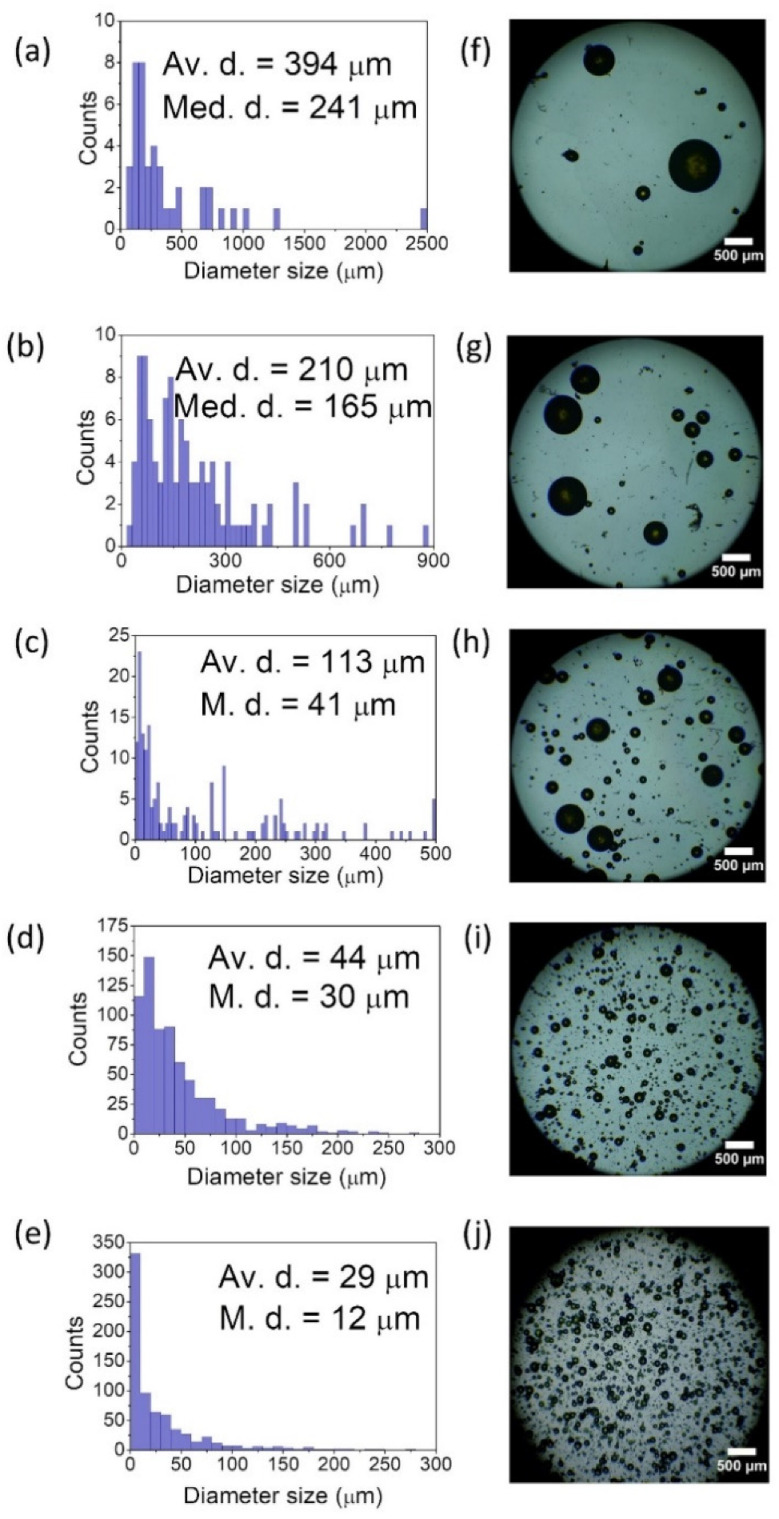
The size distribution of created LC droplets inside PVA matrix and the example of microscopic pictures for emulsions homogenized for (**a**,**f**) 5 min, (**b**,**g**) 10 min, (**c**,**h**) 20 min, (**d**,**i**) 30 min, (**e**,**j**) 60 min. Abbreviations: Av. d.—average diameter size, M. d.—median diameter.

The PER material of interest, which proved perfect compatibility with various soft-matter matrices, has led to the construction of a blend, an emulsion structure that combines the properties of both the polymers and the LCs. Development of the emulsion demands the creation of a two-phase dispersion system of two mutually incompatible liquids, polar and nonpolar [56]. The only parameter that was varied during the experiment was the homogenization time of the emulsion mixture in the ultrasonic water bath (temperature set for 60 °C). We prepared five mixtures containing the LC-PER in PVA emulsion. For clarity of this article, starting from this point, the samples will be denoted samples 1 (5 min homogenization), 2 (10 min), 3 (20 min), 4 (30 min), and 5 (60 min). The obtained results, including the statistical distribution of droplet size and the average and median diameter, are presented in Figure 6a–e.

The averaged diameter size examined with a help of ImageJ software shows a decreasing trend with increasing homogenization time. The tendency starts from 394 μm for sample 1, going through 210 μm for 2, then passing by 113 μm for 3 and 44 μm for sample 4, and finishing with 29 μm for sample 5. A similar trend is visible for the other parameter indicated here, which is the median diameter (241, 165, 41, 30, and 12 μm, respectively). The corresponding pictures (Figure 6f–j) made with the help of an optical microscope present the droplets’ shape, size, and distribution. At this stage, it is worth paying attention to sample number 5. The result of the average droplet size and the median can be considered overstated. During close observation of the microscopic photos, one can see many droplets characterized with a diameter below 1 micrometer. Therefore, it is not possible to specify and analyze their exact number. The structure of sample 5 contains visible droplets of a dozen or tens of micrometers. However, in the matrix, a large part of the LC material has been efficiently homogenized, creating a system similar to milk or mayonnaise. In this case, it is not possible to list all the suspended droplets, so the given diameter and median values are erroneous.

**Figure 7:**
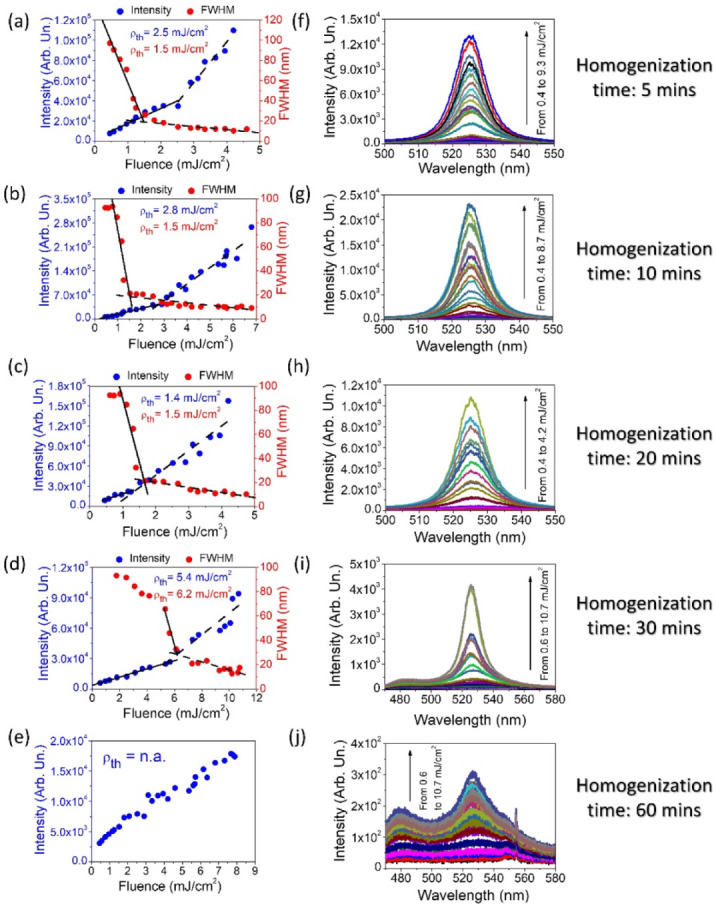
The stimulated emission analysis for different times of emulsion homogenization. The PER dye concentration is equal to 0.4% for all samples and the energy thresholds are designated for the varied homogenization time: (**a**) 5, (**b**) 10, (**c**) 20, (**d**) 30, and (**e**) 60 min. The spectra correlated with the estimated STE $\rho_{\mathrm{th}}$ are presented in (**f**–**j**).

By observing the number of formed droplets and being aware that each sample contains the same amount of PER/LC mixture, one can confidently hypothesize that the longer homogenization time is, the more resonators are obtained. Light amplification studies led to the comparison of designated STE energy thresholds (see Figure 7a–e). The corresponding spectra are presented in Figure 7f–j. 

For this type of matrix, the STE energy threshold analysis was based on the two afore-discussed methods, namely FWHM and LI-LO. With the use of FWHM and LI-LO, sample 1 shows energy thresholds for the STE phenomenon at the values of 1.5 and 2.5 mJ/cm^2^, respectively. Sample 2 is characterized with very similar results for this approach analysis, namely 1.5 and 2.8 mJ/cm^2^. At this point, it can be noted that the change in the average size of the resonators does not cause too much discrepancy in the energy threshold values. Going further, for sample number 3, the thresholds determined for both methods are very similar and amount to about 1.5 mJ/cm^2^. It is worth commenting on sample number 4, in which the STE energy threshold is noticeably higher. Using the LI-LO method, it is indicated at 5.4 mJ/cm^2^. When analyzing the spectral half-width changes, it is visible that this value has decreased for the energy density of 6.2 mJ/cm^2^. Presumably, there are many smaller diameter resonators in the sample. After half an hour of the homogenization process, the LC/PER material splits into fine droplets; their average size is estimated at 44 μm. The emulsion layer visually looks much more consistent in terms of the droplet size distribution (Figure 6i). In the case of this emulsion, a very good log-normal fit can be distinguished with the R^2^ parameter equal to 93% (Figure S5). Thus, the sample obtained a good degree of homogenization and compliance with the log-normal distribution, which is often a model for spontaneously emerging structures within a given study population. The low STE energy threshold should be correlated with the diameter of the resonators. Apparently, the light needs a longer path to be amplified efficiently, which was obtained in samples 1, 2, and especially 3. The compatibility with log-normal distribution was also found in sample 2, although with a significantly lower R^2^ value (75%). 

**Figure 8:**
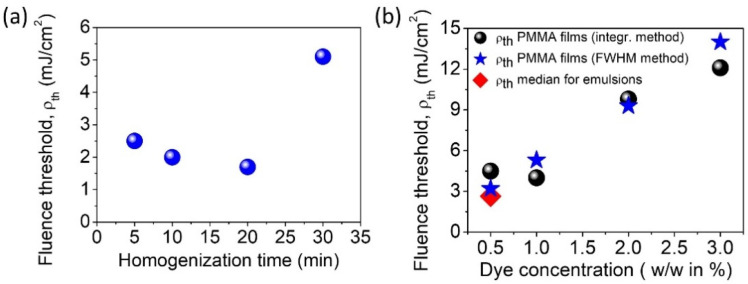
(**a**) The correlation between emulsion STE energy threshold and homogenization time. (**b**) The STE $\rho_{\mathrm{th}}$ of PMMA thin films as a function of dye concentration, compared with the median value of the threshold for LC emulsions.

The results of light amplification in sample number 5 are very interesting. It is impossible to find a narrowing of the half-width of the spectra (Figure 7j) or a characteristic inflection that proves the achievement of the stimulated emission energy threshold (Figure 7e), even if pumping energy reaches 10.7 mJ/cm^2^. Additionally, no log-normal match (Figure S5e) was found. From the previous analysis, it is known with certainty that sample 5 was homogenized for the longest time. In the sample volume, many small optical resonators are preventing the light from being amplified, according to the fact that nonconstructive scattering in the sample dominates over the constructive one, not allowing the effect of stimulated emission to be obtained. Too high losses related to the photons scattering without amplification effect are recorded in this case. Moreover, sample 5 resembles a chaotic system since larger resonators are also distinguished, similar to the ones from samples 1–4. However, the measurement of STE is averaged, and its result is influenced by many micrometer resonators scattered randomly in the matrix. Hypothetically, we can assume that these droplets are too small to effectively amplify the light, so they act as diffusers and significantly affect the negative result of obtaining STE. To conclude, since the sample is homogenized longer, it results in smaller droplets (here, identical to the resonators). However, this does not mean that at each point of homogenization we can obtain a satisfactory level of matching to the log-normal model. Since homogenization takes place spontaneously, the appearance of similar droplet sizes should not be expected at every point, and the tendency is not linear to the time of mixing. In Figure 8a, we present the correlation between emulsion STE energy threshold and time of homogenization. 

It is clearly presented that with the homogenization time of 30 min one should expect a higher energy threshold, according to the significant reduction in droplet diameter size. In samples 1–3, the results are slightly different. The observed differences are not very large, probably due to the light amplification effect obtained by averaging (the 4 mm long laser light beam in strip geometry was used). Figure 8b visualizes all the STE energy thresholds for the PMMA thin films indicated here, compared to the median value of the LC emulsion threshold. For the 0.5% PER dye concentration, a lower energy threshold is observed for the median of emulsions (about 2.6 mJ/cm^2^), and the highest one is indicated for PMMA films (LI-LO method, 4.6 mJ/cm^2^). However, by recalling the LC cell STE $\rho_{\mathrm{th}}$ thresholds, we can summarize that all of the values are sustained in the same order of magnitude. It is worth considering the differences resulting from the use of various organic matrices. The perylene derivative, serving as a dopant in the PMMA matrix, creates well-characterized, simple systems in which one can easily notice the concentration dependence on the obtained energy threshold of STE (Figure 8b). The simplicity in preparation and working of such a system is appreciated, together with the fact that the polymeric matrix does not quench the STE provided by the PER dye. Among the undisputed advantages of using PMMA matrices for laser systems, one should include inexpensive and simple production process, ease of management, nontoxicity, lack of flammability, restraint of flow fluctuations, and lower laser damage threshold in comparison with liquids [57]. In addition to these features, there is the possibility of quick and simple modulation of the PER concentration, which gives easy-to-predict information about the needed value of pumping energy. At this stage, easy modulation with chromophore concentration and the clear response of stimulated emission performed in the comparative regime can also be noted. 

Surprisingly, PER fits perfectly into the LC nematic mixture, creating a simple system of LC cells, easily modulated by small values of DC voltage. This effect depends on the LC cell thickness, the dye concentration, and the value of the applied voltage, which can be used to modulate the STE effect. For the future, much more combined systems can be created—for example, an experiment with the change in the orientation of the LC molecules from the homeotropic to the planar regime. As already mentioned, the LC environment is favorable to the formation of luminous j-type aggregates. The investigation of this particular chromophore in the aggregated version allows for obtaining ultra-photostable systems emitting amplified light [42]. In this work, it has been proved that the guest–host system using LC/PER is an excellent example of a laser device, easy to modulate and allowing for many future combinations. Such an LC cell can be easily adapted to the desired application through the thickness or low-magnitude external field influence.

The last and the most complicated emulsion system using both LC and polymer matrix (PVA) allows for the creation of optically active round-shape resonators with various diameters. Excellent phase separation and a satisfactory shape of the LC PER-doped droplets were obtained. Their size, dispersion, and ability to control these parameters make this a very interesting approach. This is a kind of alternative to the classic LC cell, as it assumes the creation of a specific size of resonators and examines the influence of their geometry on the phenomenon and strength of the obtained STE. An interesting area for future investigation is the testing of STE parameters for single droplets, varied by diameter size. However, for STE investigation, the spontaneous process of the emulsion formation is also important in the context of understanding and controlling the specific population of droplet resonators in the device volume. Creating a transition between the STE- emitting (samples 1–4) and the STE-quenching (5) droplets could be an important step in the development of easily switchable LC-based organic lasers or lasers (emitters) serving as mechanical sensors, the emission of which is strongly sensitive to external shocks. For this reason, a suitably compatible and reliable chromophore is needed, which in this case has undoubtedly been found.

## 4. Conclusions

In summary, we have reported different laser devices based on a chosen optical gain material, perylene derivative (PER). The chromophore, used to dope different organic matrices (PMMA, LC nematic mixture 1742, LC nematic structure 5CB in PVA matrix), shows remarkable compatibility and effective tunability possibilities, imposed by different stimuli. On top of this, we have found the effective STE effect and different ways of enhanced emission tunability in all investigated systems. Enhanced emission tunability was realized via changing the thickness of the LC cell, varying the concentration of the added PER dye, selecting different matrices (hydrophilicity or hydrophobicity), applying various resonator sizes, employing different system constructions, and testing the applied electric field. The results reported in this contribution represent the essential step for control of stimulated emission character and obtaining a variety of effects with only one active versatile dye and easily available, compatible organic materials. The work is dedicated towards novel laser technologies that require (i) simple, concentration-tunable light-emitting systems (PER/PMMA) dedicated to emission switching; (ii) externally influenced systems (LC cells with PER), or (iii) miniaturized laser light sources (PVA emulsions with LC/PER microresonators).

## Data Availability

Not applicable.

## Supplementary Materials

The following supporting information can be downloaded at: https://www.mdpi.com/article/10.3390/ma15030980/s1, Figure S1: ^1^H NMR spectra of PER, Figure S2: ^13^C NMR spectra of PER, Figure S3: DEPT-135 NMR spectra of PER, Figure S4: The maximum intensity of the gradient sample as a function of LC cell thickness (arrow guided by eye) (a) and the schematic presentation of the performed experiment (b), Figure S5: The log-normal distribution for all investigated emulsions homogenized for (a) 5 min, (b) 10 min, (c) 20 min, (d) 30 min, and (e) 60 min.
