# Supplementary material for: Perylene-Based Chromophore as a Versatile Dye for Light Amplification
Source: Materials (Basel). 2022 Jan 27;15(3):980. doi: 10.3390/ma15030980 (PMC8838310; doi:10.3390/ma15030980)
Supplement: Supplementary file 1 [file materials-15-00980-s001.zip › materials-1489849-supplementary.pdf]

Supplementary materials

Perylene-Based Chromophore as a Versatile Dye for Light Amplification

Alina Szukalska [1], Adam Szukalski [1], Justyna Stachera [2], Dorota Zając [1], Ewa Chrzumnicka [2] ,Tomasz Martynski [2] and Jaroslaw Mysliwiec [1]

[1] The Advanced Materials Engineering and Modelling Group, Faculty of Chemistry, Wroclaw University of Science and Technology, Wybrzeze Stanislawa Wyspianskiego 27, 50-370 Wroclaw, Poland; adam.szukalski@pwr.edu.pl (A.S.); dorota.zajac@pwr.edu.pl (D.Z.); jaroslaw.mysliwiec@pwr.edu.pl (J.M.)

[2] Faculty of Materials Engineering and Technical Physics, Poznan University of Technology, Piotrowo 3, 60-965 Poznan, Poland; justyna.m.stachera@doctorate.put.poznan.pl (J.S.); ewa.chrzumnicka@put.poznan.pl (E.C.); tomasz.martynski@put.poznan.pl (T.M.)

* Correspondence: alina.szukalska@pwr.edu.pl; Tel.: +48713203197

S1. ¹H NMR spectra

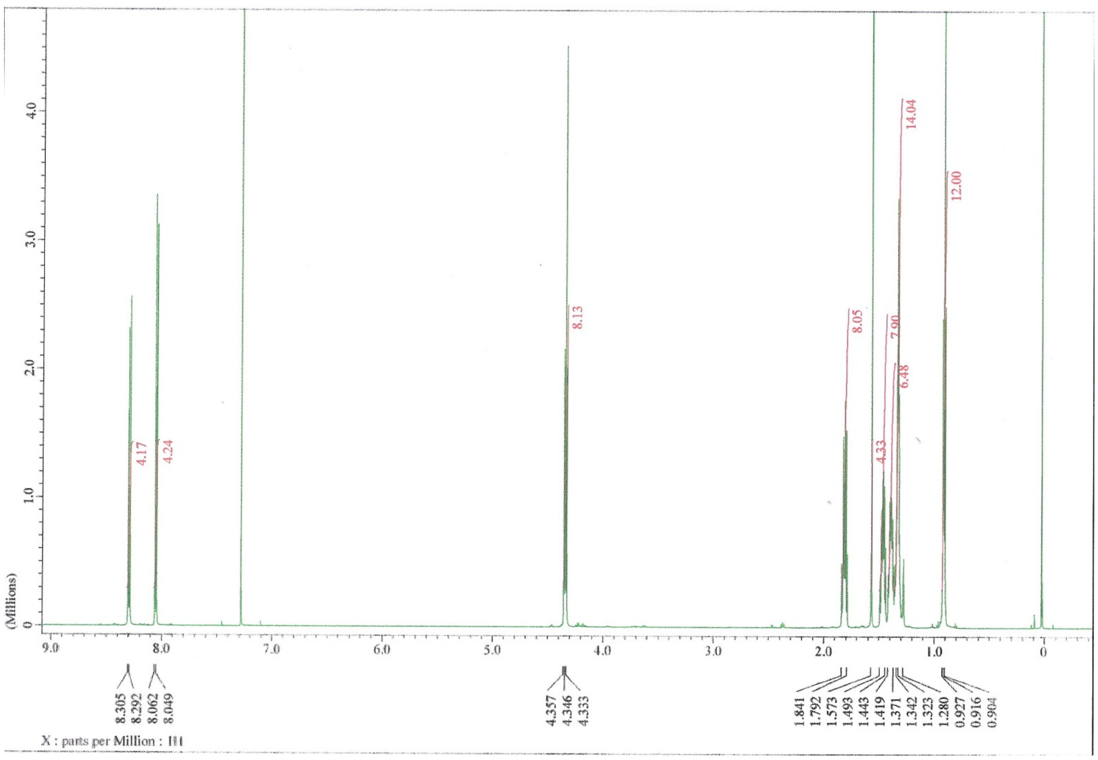

Figure S1. ^{1}H NMR spectra of PER.

S2. ^{13}C NMR spectra

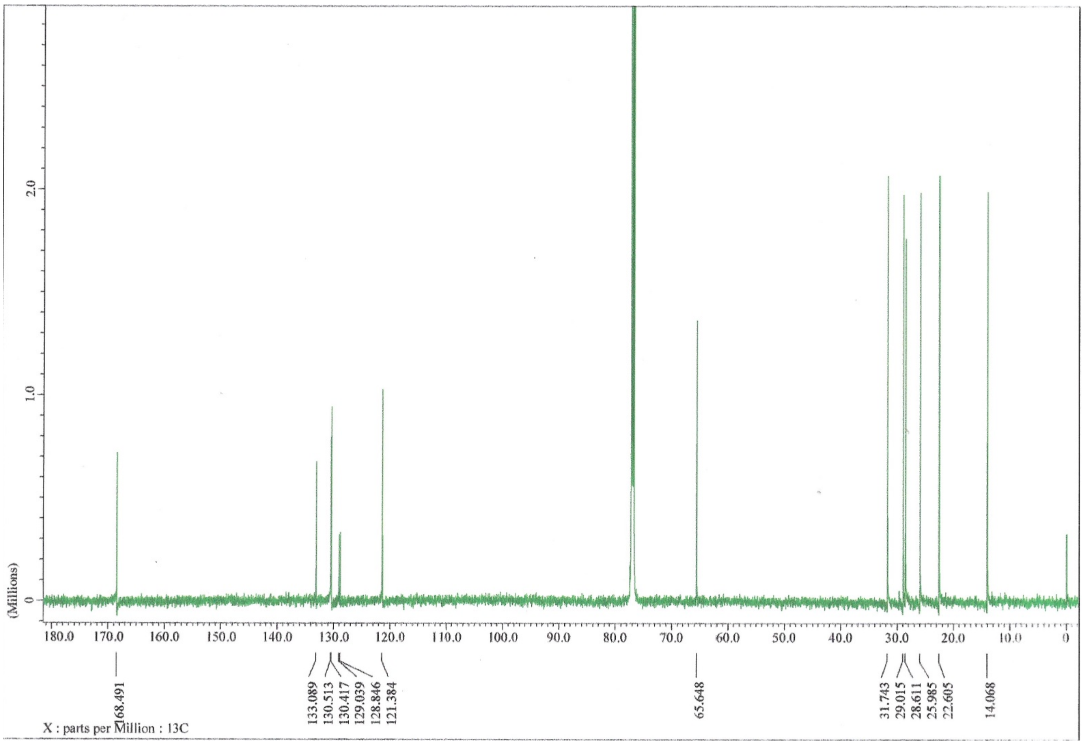

Figure S2. ^{13}C NMR spectra of PER.

S3. DEPT-135 NMR spectra

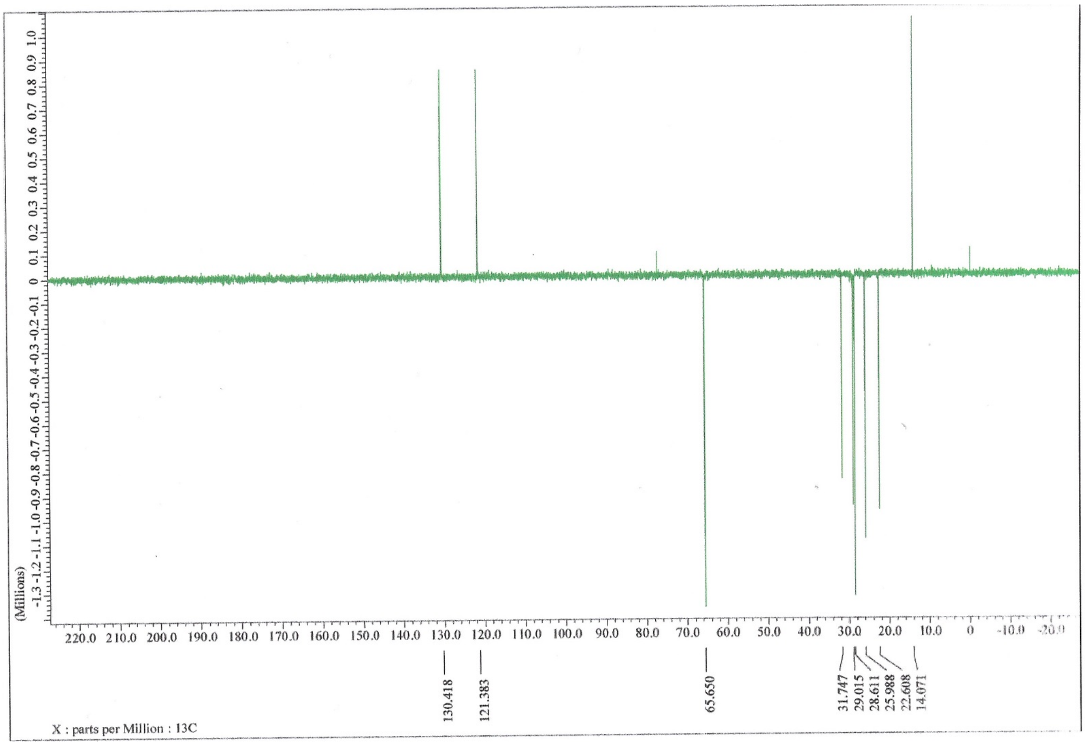

Figure S3. DEPT-135 NMR spectra of PER.

S4. Gradient thickness LC cell – optical gain insight

Since the thickest 5CB LC cell (d = 60 μm) doped with perylene dye was already investigated recently by us [25], the current approach considers the influence on the gradient cell thickness construction for its spectroscopic features, especially the optical gain

property (Figure S1). As far, as we know from the experience [25] and also from this article, where LC cell thickness is relatively thin (d = 20 μm), we decided to characterize all these sample regions, where the soft matter (LC) is non-covalently mixed with perylene laser dye and placed in the thinnest region, but with continuously increasing thickness up to the middle zone of the cell (between 20-36 microns). Our approach assumed the investigation of the output light amplification intensity and energy threshold values determining efficient optical gain activation (Figure S1). In particular, the inducing LA phenomenon laser spot was irradiated LC cell, where its thickness was equal to: 22, 25, 28, 32, and 35 microns. As far as we achieved all these experiments we found that the optical gain efficiency is increasing together with the higher LC cell thickness (Figure S1(a)). However, after a certain value of the optical path (in here, LC volume with embedded laser dye), the thickness seems to has less impact on the output intensity level. It is in agreement with the results for the spectroscopic investigation on the middle region of the gradient LC cell shown in this article (Figure 4), and our previous investigation with almost two times thicker sample (d = 60 μm). The performed trials brought a conclusion determining light amplification efficiency with LC cell thickness, indicating a significant increase when the thickness is changing and is placed in the region below 30 microns, and noticeable saturation level above 60 microns thickness. Such observation can lead to another approach to LA output energy control, related straightforwardly to the sample construction without changing other crucial parameters, like laser dye concentration.

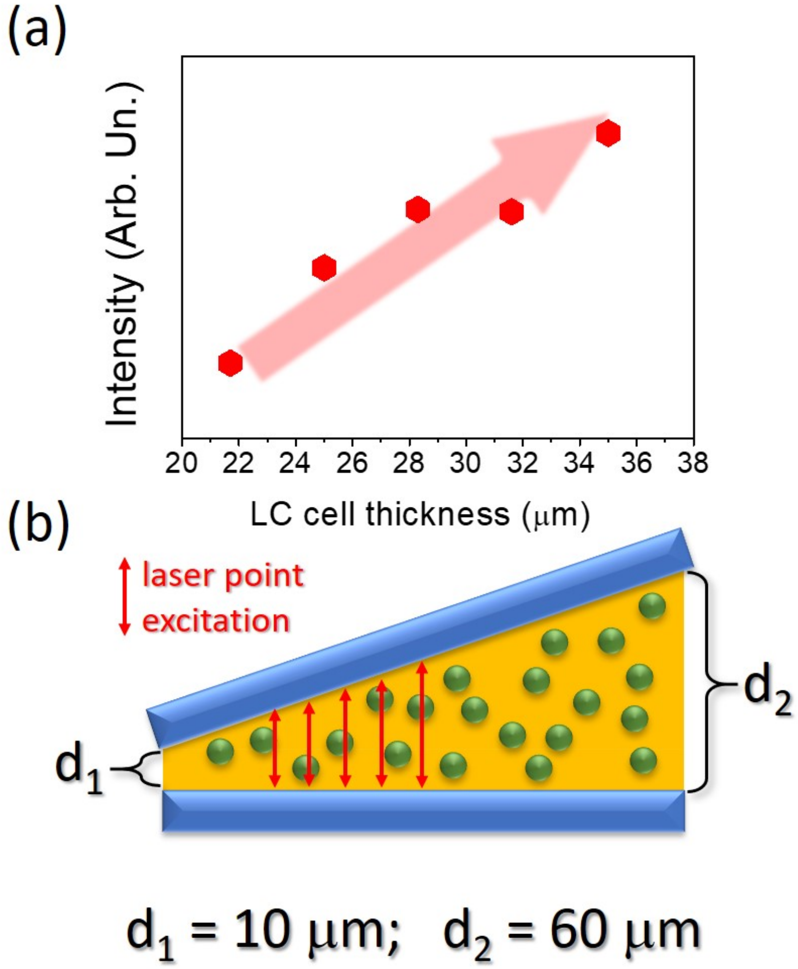

Figure S4. The maximum intensity of the gradient sample in the function of LC cell thickness (arrow guided by eye) (a) and the schematic presentation of the performed experiment (b).

S5. PER dye in emulsion – statistical approach

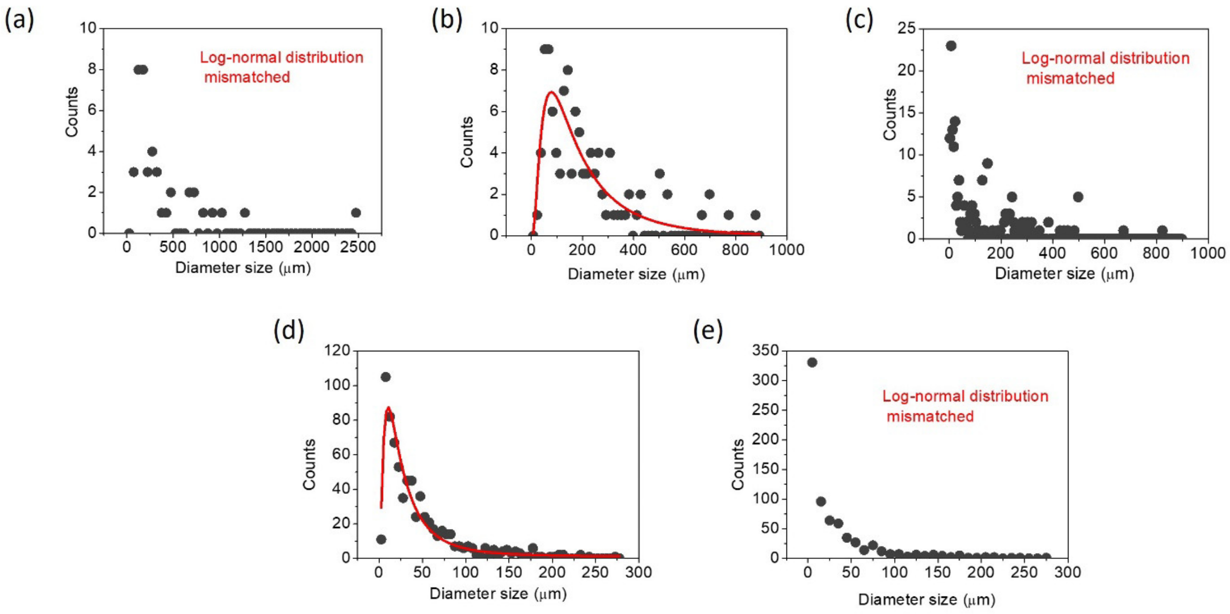

Figure S5. The log-normal distribution for all investigated emulsions homogenized for (a) 5 minutes, (b) 10 minutes, (c) 20 minutes, (d) 30 minutes and (e) 60 minutes. .
